# Glial and immune dysregulation in glaucoma independent of retinal ganglion cell loss: a human post-mortem histopathology study

**DOI:** 10.1186/s40478-025-02066-0

**Published:** 2025-06-28

**Authors:** Akanksha Salkar, Viswanthram Palanivel, Devaraj Basavarajappa, Mehdi Mirzaei, Angela Schulz, Peng Yan, Vivek Gupta, Stuart Graham, Yuyi You

**Affiliations:** 1 Macquarie Medical School, Faculty of Human, Health, and Medical Science, Macquarie University, Sydney, NSW Australia; 2 Save Sight Institute, University of Sydney, Sydney, NSW Australia; 3 Department of Ophthalmology & Vision Sciences, University of Toronto, Kensington Eye Institute/UHN, Toronto, Canada

**Keywords:** Glaucoma, Retinal ganglion cell, Gliosis, Microglia, Blood‒retinal barrier

## Abstract

Glaucoma is characterized by progressive retinal ganglion cell (RGC) loss and optic nerve head (ONH) changes, but the roles of glial activation and immune responses remain unclear. This study examines gliosis, microglial diversity, and inflammation in postmortem retinal tissues. Postmortem retinal and ONH samples (total *n* = 50) from patients with open-angle glaucoma (G, *n* = 18) were compared with those from age-matched controls (*n* = 32), including healthy individuals (Ctrl) and disease controls (patients with early age-related macular degeneration [AMD] and diabetes mellitus [DM]). Immunostaining was performed to assess glial activation, blood–retinal barrier (BRB) integrity, and immune infiltration, which were quantified via ImageJ and Zen lite. Generalized estimating equations (GEEs) with Bonferroni correction accounted for intrapatient variability. G retinae presented significant RGC loss accompanied by widespread gliosis, with activation of microglia (Iba1), astrocytes (GFAP), and Müller cells (Vimentin). This gliotic response differed across conditions, with astrocyte activation being more prominent in DM and microglial activation predominating in AMD. In glaucoma, gliosis is evident even in early-stage disease, regardless of the severity of retinal ganglion cell (RGC) loss or structural changes in the ONH. Furthermore, microglia showed a marked shift in morphological diversity, transitioning to hyperramified, bushy, and amoeboid forms, along with an increased distribution of activation markers such as CD45, CD11b, and CD163. Additionally, biochemical evidence of alterations to the BRB integrity, characterized by reduced tight junction protein expression, facilitates immune cell infiltration, as indicated by the minimal and inconsistent presence of CD3/CD4+ T cells. Gliosis persisted regardless of RGC loss severity, suggesting that gliosis progresses independently of neuronal degeneration. Unlike AMD and DM, where specific glial subtypes dominate, glaucoma exhibits widespread gliosis. Microglial heterogeneity indicates the existence of a continuum of functional states. Furthermore, dysregulation of the BRB, inconsistent immune infiltration, and multimodal microglial activation indicate that the inflammatory response in glaucoma patients is driven primarily by resident microglia, with limited interactions with infiltrating immune cells. These findings highlight the need for further research into glial modulation as a potential therapeutic strategy.

**Supplementary Information:**

The online version contains supplementary material available at 10.1186/s40478-025-02066-0.

## Background

Glaucoma, a neurodegenerative disease, is the second leading cause of blindness worldwide. It involves the progressive loss of retinal ganglion cells (RGCs) and has been associated with different complex mechanisms extending beyond intraocular pressure (IOP) and age [1]. Despite extensive research, the exact mechanisms underlying glaucoma pathology remain incompletely understood [2]. Neuroinflammation, defined as the response of activated glial cells, has been proposed to play a role in glaucoma progression. However, the specific roles and interactions of glial cells in glaucoma remain unclear [3–5].

The retinal glial cell population, comprising microglia, astrocytes, and Müller cells, is critical for maintaining retinal health and function. Microglia serve as immune sentinels [6, 7]. Astrocytes support neuronal metabolism and the blood‒retinal barrier (BRB) [8, 9], and Müller cells regulate the extracellular environment and neuronal function [10]. Dynamic interactions between these glial cells influence both retinal homeostasis and disease progression. Studies in animal models have extensively reported significant morphological and functional changes in these glial cell populations in response to glaucomatous degeneration [11–16]. Upon activation, glial cells undergo morphological and functional diversification. These reactive glial cells could either be protective or detrimental, depending on the specific microenvironmental stimuli. Although reactive changes in glial cells have been demonstrated, detailed characterization, particularly in human tissues, remains limited [17–20].

The retina is immune-privileged due to the BRB; disruption of this barrier under pathological conditions can allow systemic immune cell infiltration, potentially exacerbating inflammatory responses [21]. BRB dysregulation has been studied in animal models, although data from human tissues remain limited, highlighting the need for further exploration [19]. Furthermore, immune infiltration or increased RGC loss due to the adoptive transfer of immune cells has been shown in animal models [14, 22]. These studies have revealed the active role of systemic immune components in glaucoma pathology. However, the precise contribution of immune infiltration to human glaucoma remains to be clarified.

To address these gaps, we analysed postmortem retinal samples from glaucoma patients, control individuals, and patients with other retinal diseases, such as age-related macular degeneration (AMD) and diabetes mellitus (DM). To distinguish glaucoma-specific alterations from changes common to other retinal or systemic diseases, AMD and DM cases were included as disease controls. Our study comprehensively assessed RGC loss, extracellular matrix (ECM) protein alterations, glial cell dynamics, microglial activation, BRB changes, and immune cell infiltration, providing an integrated view of retinal changes associated with glaucoma in human tissues. While the findings are associative, they offer valuable insights into potential mechanisms underlying disease progression.

## Methods

### Sample collection and preprocessing

Formalin-fixed left and right eye globes from donors (primary open-angle glaucoma (G), age-matched healthy individuals (Ctrl), age-related macular degeneration (AMD) and diabetes mellitus (DM)) were acquired from Lions NSW Eye Bank. Human ethics approval was obtained for these samples (Ref No. 52023319446659). All donors consented to the use of tissue for research purposes in compliance with ethical standards. The early AMD samples showed no signs of geographic atrophy or choroidal neovascularization, and the samples from DM patients did not exhibit diabetic retinopathy. These samples were chosen to minimize interference from advanced disease stages in our findings. The samples were stored in preservation solution (formalin, median time in preservation solution = 1.20 ± 0.05 years). The formalin-fixed eye globes were dissected sagittally, and retinal strips (~ 5 mm wide) extending from the optic disc to the ora serrata were dissected from the superior and inferior quadrants. The dissected strips were then embedded in paraffin and sectioned into 7 μm-thick sections via a microtome (Mikrom HM325 Microtome). Three sections were mounted on each slide.

### Histological analysis

**Fig. 1:**
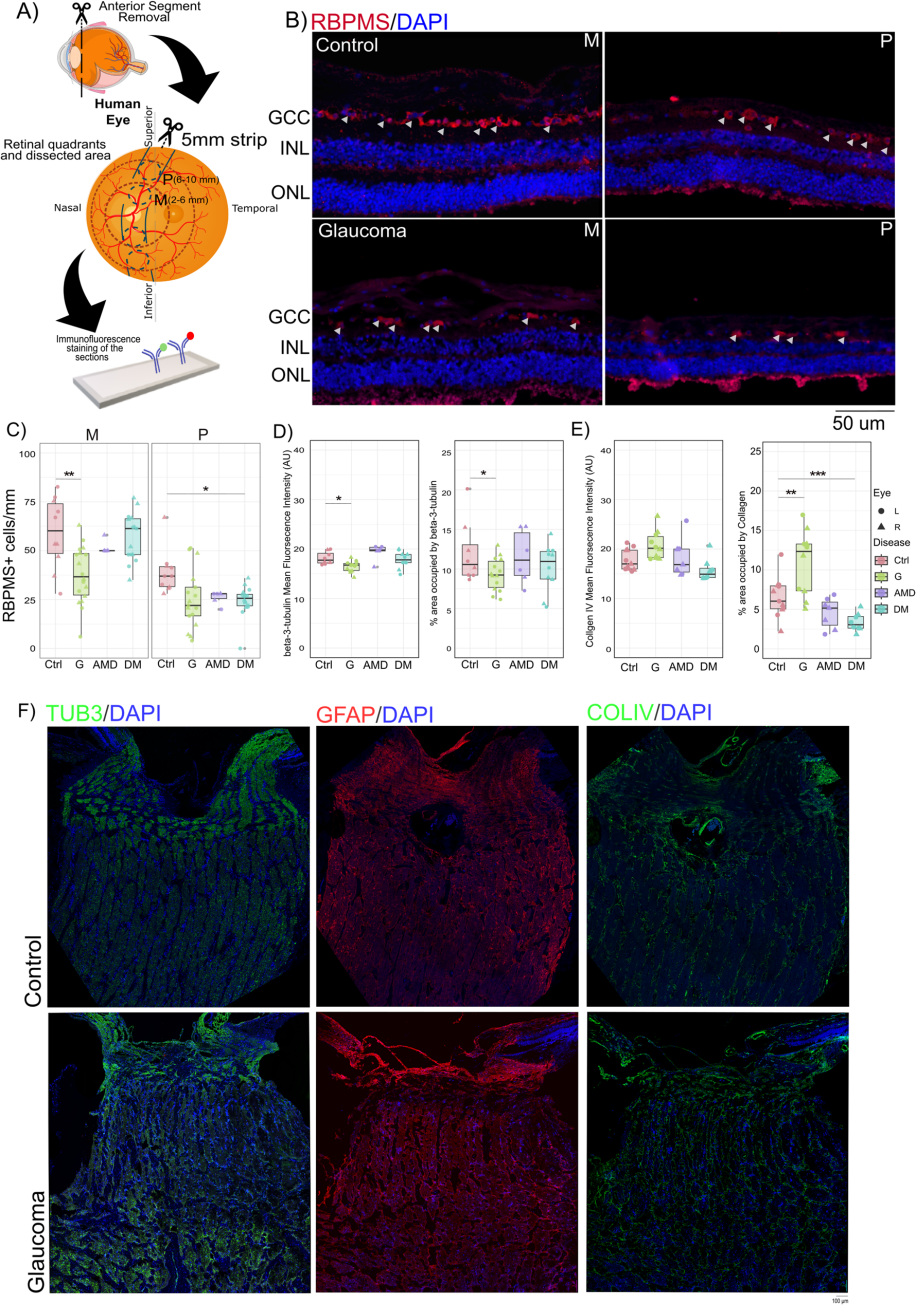
Retinal ganglion cell (RGC) loss and changes in the optic nerve head (ONH). (**A**) FFPE retinal sections were analysed to examine retinal and ONH changes in glaucoma, AMD, and DM samples. (**B**) Representative images showing RBPMS-stained RGCs in the mid-peripheral and peripheral retinal regions in the G and Ctrl groups. (**C**) RGC loss was observed in both the mid-peripheral and peripheral retinal regions in glaucoma samples, unlike in AMD and DM samples, as visualized via a boxplot. There was also a significant reduction in beta-3-tubulin staining intensity and percentage area occupied, indicating decreased axonal density (**D**). Additionally, an increase in ECM accumulation was observed, as measured by elevated collagen IV staining (**E**). (**F**) Representative images highlighting differences in beta-3-tubulin, GFAP, and collagen IV staining between control and glaucoma samples. Abbreviations: FFPE = formalin-fixed paraffin-embedded; AMD = age-related macular degeneration; DM = diabetes; GCC = ganglion cell complex; INL = inner nuclear layer; ONL = outer nuclear layer; Ctrl = control; G = glaucoma; M = mid-peripheral; P = periphery. Symbol Meaning: * *P* ≤ 0.05; ** *P* ≤ 0.01; *** *P* ≤ 0.001; **** *P* ≤ 0.0001

Before immunostaining, the sections were deparaffinized and rehydrated via an ethanol gradient. Antigen retrieval was performed via microwave-assisted heat-induced epitope retrieval in 10 mM sodium citrate buffer containing 0.1% Triton X-100 (pH 6.0) for 30 min at 30% (200–300 watts) microwave power. The slides were then blocked for 2 h at room temperature with blocking buffer (5% donkey serum in PBS with 0.3% Triton X-100). The slides were incubated with primary antibodies overnight at 4°C [23]. A list of the primary antibodies used is provided in Supplementary Table 1. The slides were washed three times with 1X PBS for 5 min each and then incubated with secondary antibodies and DAPI (Supplementary Table 1). The slides were sealed with antifade mounting medium. (Fig. 1a). We also maintained negative controls, omitting primary antibodies, to confirm the absence of nonspecific staining. To control for donor-specific autofluorescence, unstained sections from each donor were imaged using identical settings. Antibody dilutions were optimized via practice runs.

### Image acquisition and analysis

The slides were then observed a ZEISS Axio Imager 2 fluorescence microscope (ZEISS, Carl Zeiss, Oberkochen, Germany). All the images were acquired under identical settings within the dynamic range by optimizing the imaging parameters, including the laser intensity, gain, and exposure time. The retinal strips were divided into two subregions on the basis of the distance from the optic nerve head (ONH): the mid-periphery (M; ~2 mm from the ONH) and the periphery (P; ~6 mm from the ONH). Two images were acquired at each point from both sides of the ONH at 20x and 40x magnification. This resulted in 12 images per slide. The ONH images were captured at 40x magnification via a tiling technique, with the individual tiles merged to create a complete image.

### Image analysis

Images from each channel were also exported as separate images. ImageJ was used to calculate the relative fluorescence intensity and % area of the retina, and ONH was immunoassayed.

### Relative fluorescence intensity and % area covered

#### Relative Fluorescence intensity and %area covered

The image for the appropriate channel was converted to 8-bit TIFF format. Regions of interest (ROIs) were applied to cover the entire retinal cross-section. Background subtraction was performed via the rolling ball method (radius = 50). The threshold was adjusted for all the images. The relative intensity and % area covered were then measured for all the images, and values at M and P were averaged. These processing steps were automated via an ImageJ macro to maintain reproducibility.

### Blood vessel staining

Images of 10–15 blood vessels per sample were acquired under consistent imaging conditions. Only vessels that were not distorted during processing were included in the analysis. Vessels were systematically selected from the corresponding M, P, and ONH subregions across all the samples to ensure uniformity. Fixed ROIs were applied to each sample, and the relative intensity was calculated via a standardized method, as previously described, employing an ImageJ macro for image processing.

### Cell counts

For cell counts, images were acquired at the M and P retinal regions. Two images were acquired at both points in each section. Cell counts were performed manually within Zen Lite. The cell counts were normalized to 1 mm of the retina by measuring the retinal length along the outer nuclear layer (ONL) outer border. For RGC counts, the RBPMS + cells in the ganglion cell layer (GCL) layer were counted in the M and P regions. For microglial cell counts, cells within the following retinal layers were counted: the ganglion cell complex (GCC, comprising the retinal nerve fibre layer, ganglion cell layer, and inner plexiform layer), inner nuclear layer (INL), ONL and blood vessels. Microglia were also observed for their morphology while counting. For ease of analysis, microglial cell morphology was categorized as ramified (resting), hyperramified, bushy, or ameboid [24].

### Statistical analysis

All the quantifications performed for the four sample groups are presented as the means and 95% CIs. To address the intrapatient variability arising from the repeated measurements from both eyes of each patient, generalized estimating equations (GEEs) [25] were employed. All the statistical analyses were performed in SPSS version 29, and statistical significance was defined as *p* ≤ 0.05. Post hoc pairwise comparisons between disease groups were conducted via Bonferroni correction to control for multiple comparisons. These results are presented as the mean absolute difference, with *p* values indicating the statistical significance of differences between groups. Significant results are also reported with their respective 95% confidence intervals (CI 95%, lower-upper).

Hierarchical clustering [26, 27] was performed using the variables RGC counts, Iba1 + cell counts, Iba1 intensity, GFAP intensity and vimentin intensity. Missing values were imputed via groupwise means via R programming. The values were standardized via Z score transformation (mean 0, SD 1). Ward’s method was used to perform clustering, and Euclidean distance was used as a distance measure [28]. The cluster membership and dendrogram generated were saved. The cluster membership and disease groups were then used for cross-tabulation for classification.

Correlation analyses were performed to assess the associations between RGC counts and multiple glial markers. Pearson correlation coefficients (r) were calculated to assess the strength and direction of the associations between RGC counts and each glial parameter that was normally distributed. Moreover, Spearman’s rank correlation was calculated for nonnormal variables. Statistical significance was determined via a two-tailed *p* value threshold of 0.05. Analyses were performed via SPSS.

All plots and visualizations were generated via R statistical software (v4.1.2; R Core Team 2021).

## Results

A total of 50 postmortem eye globes were included in this study, and the participant demographics are summarized in Table 1. Figure 1a illustrates our sampling strategy, which included the optic nerve head as well as the mid-peripheral and peripheral retinal regions. We avoided the macular area to prevent AMD-related changes from confounding our glaucoma-focused analysis. All AMD samples were microscopically assessed and confirmed to be early AMD without geographic atrophy or choroidal neovascularization. All the DM samples were examined to rule out evident diabetic retinopathy.

**Table 1:** Patient characteristics

Disease Status	Number of	Gender		Age (in years)		Time in fixative (in years)	
	Eye globes (n)	Male	Female	Median	Interquartile Range	Median	Interquartile Range
Ctrl	10	60%	40%	74	54.82–81.58	1.19	1.18–1.2
G	18	77.8%	22.2%	76	70.12–82.54	2.02	1.5–2.45
DM	16	50%	50%	70.5	66.48–74.52	1.205	0.84–2.07
AMD	6	75%	25%	80	76.51–81.48	1.515	0.68–3.04

Abbreviations: Ctrl = Control, G = Glaucoma, AMD = age–related macular degeneration, DM = Diabetes

### RGC loss and glial activation might precede significant structural changes to the ONH

**Fig. 2:**
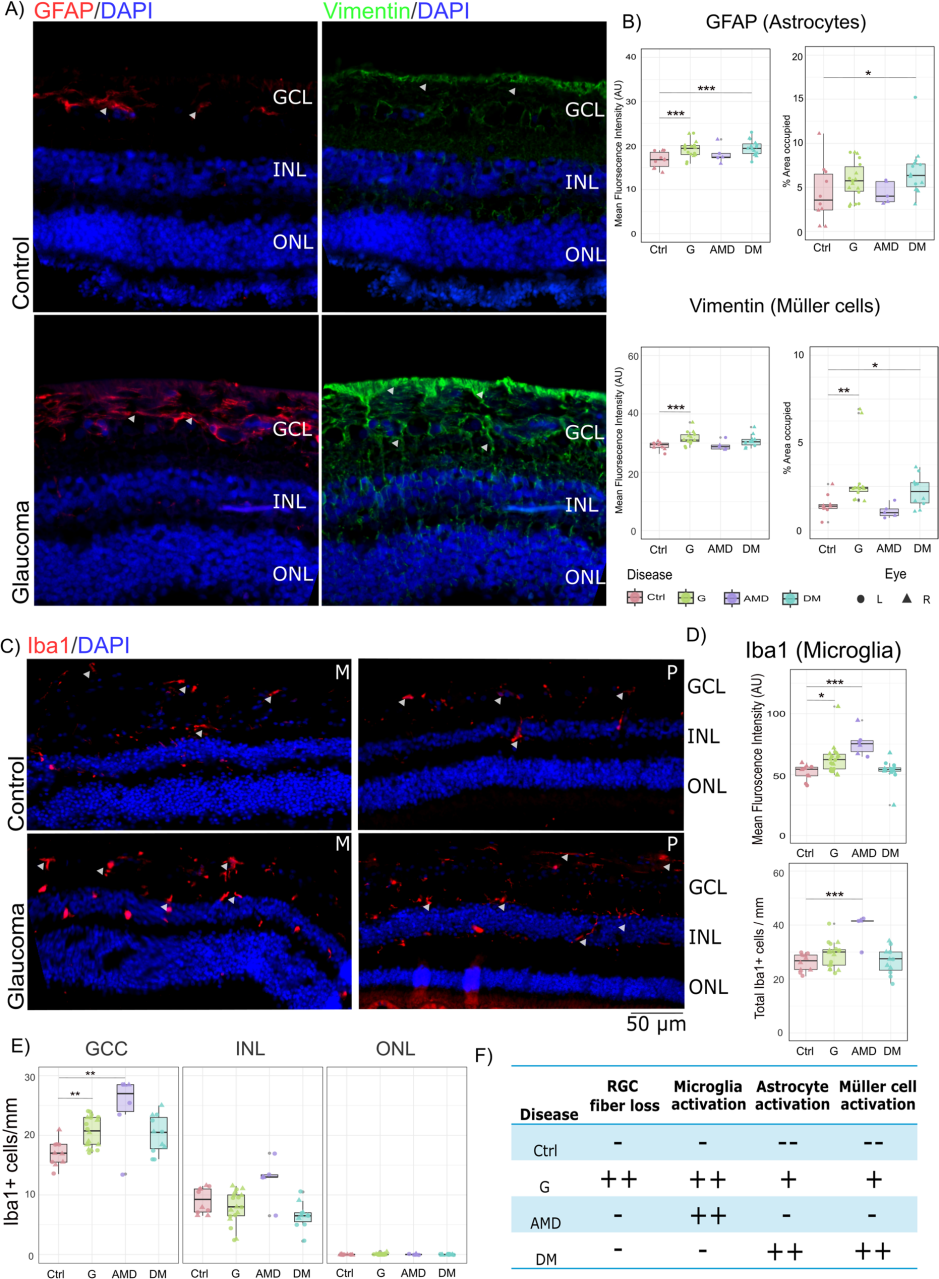
Distinct glial activation patterns in glaucoma. (**A**) Representative images showing increased activation of astrocytes and Müller cells, as evidenced by elevated GFAP and vimentin staining intensity. (**B**) Boxplots displaying the quantification of staining intensity and percentage area occupied, with significant increases observed in the glaucoma and DM samples but not in the control or AMD samples. (**C**) Representative images depict Iba1-stained microglia in control and glaucoma samples. (**D, E**) Boxplots quantifying microglial activation on the basis of Iba1 staining intensity, the total number of Iba1-positive cells (**D**), and their distribution across retinal layers (**E**). Microglial activation was significant in both the G and AMD samples. (**F**) A summary table outlines glial activation patterns across sample groups, highlighting that G exhibited macroglial and microglial activation, while AMD showed prominent microglial activation, and DM primarily displayed macroglial activation. Abbreviations: AMD = age-related macular degeneration; DM = diabetes; GCC = ganglion cell complex; INL = inner nuclear layer; ONL = outer nuclear layer; Ctrl = control; G = glaucoma; M = mid peripheral; P = periphery. Symbol Meaning: * *P* ≤ 0.05; ** *P* ≤ 0.01; *** *P* ≤ 0.001; **** *P* ≤ 0.0001

The extent of RGC loss was quantified by assessing the number of RBPMS-positive cells in the retina (Fig. 1b). As expected, a significant reduction in RGC number was observed in the G samples compared with the Ctrl and other retinal disease samples (Ctrl: mean = 49.06 cells/mm, 95% CI = 41.68–56.46; G: mean = 31.28 cells/mm, 95% CI = 26.60–36.10; *p* = 0.001). This reduction was evident across both the mid-peripheral and peripheral regions of the retina. In the mid-peripheral retina, RGC loss was still significant (Ctrl: mean = 59.10 cells/mm, 95% CI = 46.62–71.58; G: mean = 37.61 cells/mm, 95% CI = 30.71–44.51; *p* = 0.008). However, in the peripheral retina, the reduction was not statistically significant (Ctrl: mean = 39.03 cells/mm, 95% CI = 31.58–46.48; G: mean = 24.90 cells/mm, 95% CI = 15.94–33.85; *p* = 0.488) (Fig. 2c). This decrease in the number of RGCs was not detected in the other disease groups (Supplementary Fig. 1a). Most G samples exhibited structural changes in the ONH; however, the extent of ONH cupping could not be quantified due to limitations in histological sample preparation. Beta-3-tubulin staining highlighted axonal damage in RGCs at the ONH, with a modest but significant reduction in staining intensity (Ctrl: Mean = 18.15 Arbitrary units (AU), 95% CI = 17.39–18.93; G: Mean = 16.49 AU, 95% CI = 15.98–17.01; *p* = 0.003) and area occupied by the staining (Ctrl: Mean = 12.24%, 95% CI = 10.46–14.03; G: Mean = 9.48%, 95% CI = 8.63–10.34; *p* = 0.037) (Fig. 1d). Furthermore, alterations in collagen IV density were observed between Ctrl and G. There was a small but insignificant increase in collagen IV staining (Ctrl: Mean = 18.11 AU, 95% CI = 16.88–19.34; G: Mean = 20.72 AU, 95% CI = 18.86–22.59; ns); however, the area occupied by the staining increased significantly (Ctrl: Mean = 6.51%, 95% CI = 5.43–7.60; G: Mean = 10.98%, 95% CI = 8.56–13.41; *p* = 0.006) (Fig. 1e and f) (Supplementary Table 2). This increase in collagen indicates changes in the ECM in the ONH.

Next, we assessed glial cell activity through the relative staining intensity of Iba1, GFAP, and vimentin. GFAP, a marker of astrocytes, exhibited significant increase in staining intensity in G (Ctrl: Mean = 16.74 AU, 95% CI = 16.18–17.3; G: Mean = 19.17 AU, 95% CI = 18.40–19.95; *p* = 0.0001), as was vimentin, which marked the Müller cells (Ctrl: Mean = 28.89 AU, 95% CI = 27.86–29.94; G: Mean = 31.40 AU, 95% CI = 30.55–32.27; *p* = 0.002) (Fig. 2a and b). A significant increase in Iba1 intensity was detected in G retinae (Ctrl: Mean = 51.90 AU, 95% CI = 48.61–55.19; G: Mean = 62.96 AU, 95% CI = 56.52–69.41; *p* = 0.016), although the increase in the number of Iba1-positive cells was not significant (Ctrl: Mean = 26.10 cells/mm, 95% CI = 24.23–27.98; G: Mean = 28.94 cells/mm, 95% CI = 26.67–31.23; *p* = NS) (Fig. 2c and d, and Supplementary Figs. 1b and 1c). The trends in glial activity were similar in the mid-peripheral and peripheral retinal regions (Supplementary Fig. 2a, 2b, 2c, and 2d). Notably, these glial changes were observed despite the absence of significant ONH cupping, even with the inherent challenges in accurately quantifying ONH cupping.

Comparative analysis of glial activity in other retinal pathologies, such as AMD and DM, revealed distinct patterns. Compared with control samples, AMD samples presented significantly elevated Iba1 intensity, whereas GFAP levels showed some modest changes. Conversely, the DM samples presented a significant increase in GFAP intensity without a corresponding increase in Iba1 intensity (Supplementary Table 3) (Supplementary Fig. 1b and 1c). We also performed hierarchical clustering to understand how the samples clustered based on variables such as RGC counts, Iba1 intensity and numbers, GFAP intensity, and vimentin intensity. Most of the G samples clustered in cluster 2 (15/18), which was characterized by a low RGC count along with increased Iba1 intensity, Iba1 numbers, and vimentin intensity. Moreover, the samples from the other disease groups exhibited mixed clustering. Nonetheless, the samples were primarily clustered into groups with high RGC counts. AMD samples clustered in clusters 1(1), 1(1), and 4(4). Cluster 4 had moderate Iba1 intensity and number, with comparable RGC counts. Moreover, the DM samples clustered in clusters 1 and 3. Cluster 1 was characterized by high RGC counts and low glial activity, indicating normal physiology. The next cluster was cluster 3, which again had a high RGC count but moderate microglial activity and high astrocyte activity (Supplementary Fig. 2e and 2f). These findings underscore disease-specific variations in glial responses within the retina.

RGC loss, a characteristic feature of glaucoma, also reflects disease stage. To determine whether RGC loss was associated with glial activity, we examined the correlations between RGC counts and glial variables, including Iba1 intensity, microglial counts, GFAP intensity, and vimentin intensity, but found no significant relationships. Glial activity remained high regardless of RGC loss, and scatter plots revealed no distinct activation pattern linked to disease severity. (Supplementary Fig. 3 and Supplementary Table 4).

### Glial morphological alterations in glaucoma differ from those in other conditions

Gliosis also involves changes in glial cell morphology [29]. GFAP-stained astrocytes exhibited hypertrophy and extended their processes into the INL. This was marked by an increase in the area occupied by the staining in G (Ctrl: mean: 4.37%, 95% CI: 2.98–5.77; G: mean: 5.95%, 95% CI: 5.05–6.86, p = ns), although the difference was not statistically significant. Müller cells also showed a similar trend of hypertrophy and thickening of their branches, which was reflected by an increase in the area occupied by the staining (Ctrl: Mean: 1.36, 95% CI: 0.94–1.78; G: Mean: 2.91, 95% CI: 2.14–3.70, *p* = 0.004). In particular, the DM samples presented the greatest increase in the percentage of stained area for both vimentin and GFAP. (Supplementary Tables 5 and Supplementary Fig. 1b and c).

**Fig. 3:**
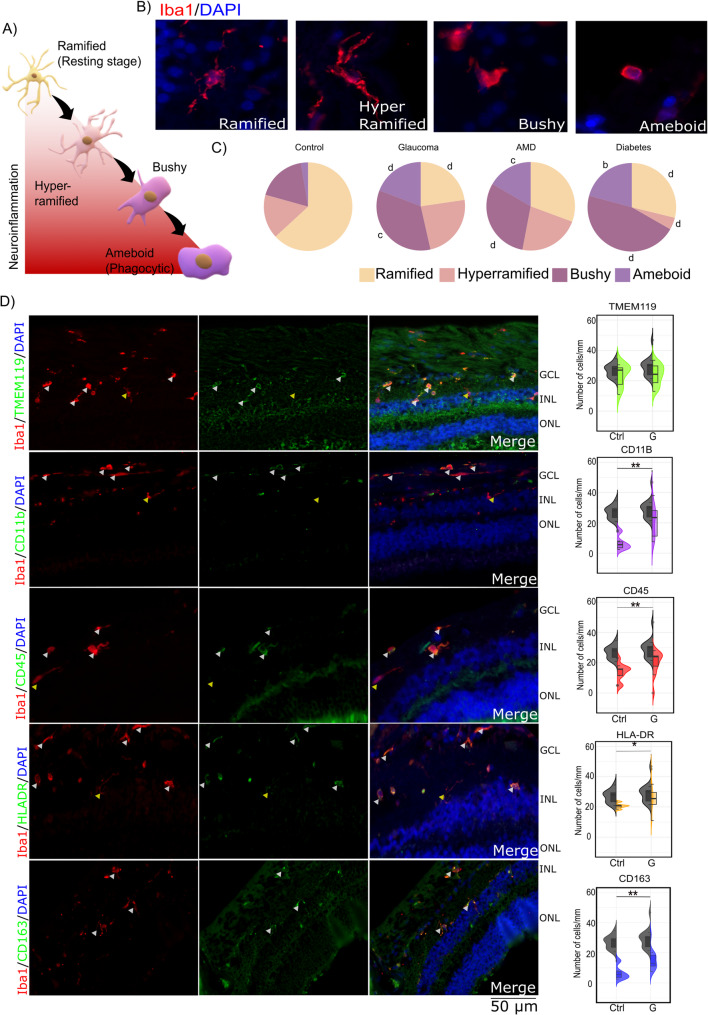
Microglia display extensive morphological and functional heterogeneity in glaucoma. (**A**) Microglia undergo morphological transformation upon activation from a resting ramified state to an ameboid phagocytic state through an intermediary bushy or ameboid state. (**B**) Representative images showing the various microglial morphologies observed in our samples. (**C**) Pie charts illustrating the shift in morphology from ramified to ameboid and other intermediary morphologies in the disease groups. (**D**) Representative images of Iba1 + microglia, along with their functional marker profiles. Split violin plots depict the distribution of marker expression in microglia. The white arrows indicate microglia positive for specific markers, whereas the yellow arrows denote marker-negative microglia. Abbreviations: AMD = age-related macular degeneration; DM = diabetes; GCL = ganglion cell layer; INL = inner nuclear layer; ONL = outer nuclear layer; Ctrl = control; G = glaucoma. Symbol Meaning: a *P* ≤ 0.05; b *P* ≤ 0.01; c *P* ≤ 0.001; d *P* ≤ 0.0001. * *P* ≤ 0.05; ** *P* ≤ 0.01; *** *P* ≤ 0.001; **** *P* ≤ 0.0001

The number of microglia increased with increasing morphological diversity under pathological conditions (Fig. 3a and b). We also observed an increase in the number of microglia in the GCC in glaucoma patients (GCC: Ctrl = 17.00 cells/mm, 95% CI = 15.49–18.52 vs. G = 20.58 cells/mm, 95% CI = 19.32–21.86 *P* = 0.051; INL: Ctrl = 9.10 cells/mm, 95% CI = 8.16–10.05 vs. G = 6.29 cells/mm, 95% CI = 6.21–9.57, P = ns); however, the increase was not significant. (Supplementary Table 6).

For ease of analysis, we divided the observed microglial morphologies into 4 categories: ramified, hyperramified, bushy, and ameboid. We observed a shift from the prominence of ramified morphology in controls to increased proportions of hyperramified, bushy, or ameboid morphology under all pathological conditions. In the controls, ramified microglia constituted 65.81% of the repertoire. However, under pathological conditions such as G, AMD, and DM, the percentages decreased to 23.16%, 32.84%, and 29.72%, respectively. All three disease groups presented an increase in the number of microglia. The AMD samples presented the greatest increase in the total number of microglia, with a significant increase in the bushy and ameboid states. Even in G, there was a significant increase in the number of bushy and ameboid microglia. In DM, there was an increase in the number of bushy and ameboid microglia, but there was also a decrease in the number of hyperramified microglia. In AMD and G patients, there were more hyperramified microglia than in controls. (Supplementary Table 7, Fig. 3c and Supplementary Fig. 4a).

### Dynamic and multimodal nature of the microglial response in glaucoma

The analysis of microglial markers revealed significant differences between control and glaucoma samples, highlighting changes in their functional and morphological states. Iba1+/CD45+ cells were significantly greater in glaucoma patients (mean: 18.50 cells/mm, 95% CI: 16.56–20.85) than in Ctrl (mean: 11.86 cells/mm, 95% CI: 8.43–15.20, *p* ≤ 0.0001), indicating heightened immune activity. Similarly, the number of Iba1+/CD11b+ cells, associated with activated microglia and macrophages, was markedly greater in G (mean: 22.54 cells/mm, 95% CI: 15.49–29.59) than in Ctrl (mean: 6.85 cells/mm, 95% CI: 3.90–9.80, *p* ≤ 0.0001). This increase was most pronounced in ramified and bushy morphologies, reflecting a reactive microglial state. The number of Iba1+/CD163+ cells was also greater in the G group (mean: 12.44 cells/mm, 95% CI: 9.05–15.82) than in the Ctrl (mean: 8.16 cells/mm, 95% CI: 6.20–10.12), particularly in ameboid cells (Table 2). In contrast, we observed low numbers of CD68/Iba1+ cells in both the retina and the ONH, although their numbers were slightly greater in the G than in the Ctrl (Fig. 3d and Supplementary Fig. 5b). Iba1+/TMEM119+ cells were not significantly different between the Ctrl (mean: 24.02 cells/mm, 95% CI: 19.51–28.53) and G (mean: 25.10 cells/mm, 95% CI: 21.88–28.32, p = NS) groups, which was consistent across microglial morphologies. Similarly, Iba1+/HLA-DR+ cells remained comparable between the Ctrl (mean: 26.21 cells/mm, 95% CI: 24.06–28.37) and G (mean: 26.93 cells/mm, 95% CI: 22.43–31.42) groups, suggesting that there was no significant alteration in antigen presentation, possibly due to para-inflammation associated with aging.

**Table 2:** Morphological and quantitative analysis of microglial activation in control and Glaucoma conditions

Markers	Control					Glaucoma				
	Total	Ramified	Hyperramified	Bushy	Ameboid	Total	Ramified	Hyperramified	Bushy	Ameboid
Iba1	27.16(23.70–30.62)	16.62(13.46–19.79)	3.96(2.88–5.05)	5.54(3.28–7.81)	0.93(0.05–1.83)	29.02(24.98–33.07)^NS^	7.09(5.33–8.84)^D^	4.88(3.30–6.45) NS	10.34(8.62–12.06)B	5.71(4.24–7.19)C
TMEM119	24.02(19.51–28.53)^ns^	16.65(11.78–21.52)^ns^	4.02(2.28–5.76)^ns^	3.88(1.50–6.25)^ns^	0.70 (0.26–1.14)^ns^	25.10(21.88–28.32)^NS, ns^	5.89(4.55–7.24)^D, ns^	6.39(4.49–8.30)^NS, ns^	8.93(7.36–10.50)^C, ns^	4.76(4.02–5.50)^D, ns^
HLA-DR	26.21(24.06–28.37)	13.97(13.33–14.60)	5.64(5.21–6.07)	9.82(7.84–11.80)	0.59(0.29–0.89)	26.93(22.43–31.42)	6.59(3.56–9.63)	4.73(2.39–7.06)	10.44(8.11–12.77)	5.60(3.53–7.66)
CD45	11.86(8.43–15.20)^d^	4.29(1.02–7.55)^d^	2.08(1.33–2.82)^ns^	3.01(1.78–4.24)^ns^	2.57(0.60–4.52)^ns^	18.50(16.56–20.85)^B, d^	3.055(2.24–3.87)^NS, d^	1.63(0.30–2.45)^NS, ns^	7.51(6.41–8.55)^D, b^	4.48(3.47–5.50)^NS, ns^
CD11b	6.85(3.90–9.80)^d^	3.61(1.38–5.85)^d^	0.94(0.17–1.72)^ns^	2.16(0.93–3.41)^ns^	2.47(0.1–5.05)^ns^	22.54(15.49–29.59)^D, ns^	3.43(1.67–5.19)^NS, a^	2.88(0.97–4.80)^NS, a^	8.14(5.79–10.50)^D, ns^	3.82(2.75–4.90)^NS, ns^
CD163	8.16(6.20–10.12)^d^	0	0	8.20(6.20–10.12)	0	12.44(9.05–15.82)^NS, d^	0.75(0.20–1.30)	5.21(3.87–6.54)	5.79(2.64–8.95)	5.56(4.57–6.54)

The data is presented as average number of cells/mm (95%CI). Abbreviations: Ctrl = Control, G = Glaucoma, AMD = age–related macular degeneration, DM = Diabetes, RGC = Retinal ganglion cell, ColIV = collagen IV, M = Mid-peripheral retina, P = Peripheral retina, GCC = ganglion cell complex, INL = inner nuclear layer, ONL = outer nuclear layer. Iba1 vs. other markers: a = *P* ≤ 0.05, b = *P* ≤ 0.01, c = *P* ≤ 0.001, d = *P* ≤ 0.0001; Ctrl vs. Disease: A = *P* ≤ 0.05, B = *P* ≤ 0.01, C = *P* ≤ 0.001, D = *P* ≤ 0.0001

### Evidence of BRB dysregulation in glaucoma and its potential role in immune infiltration

**Fig. 4:**
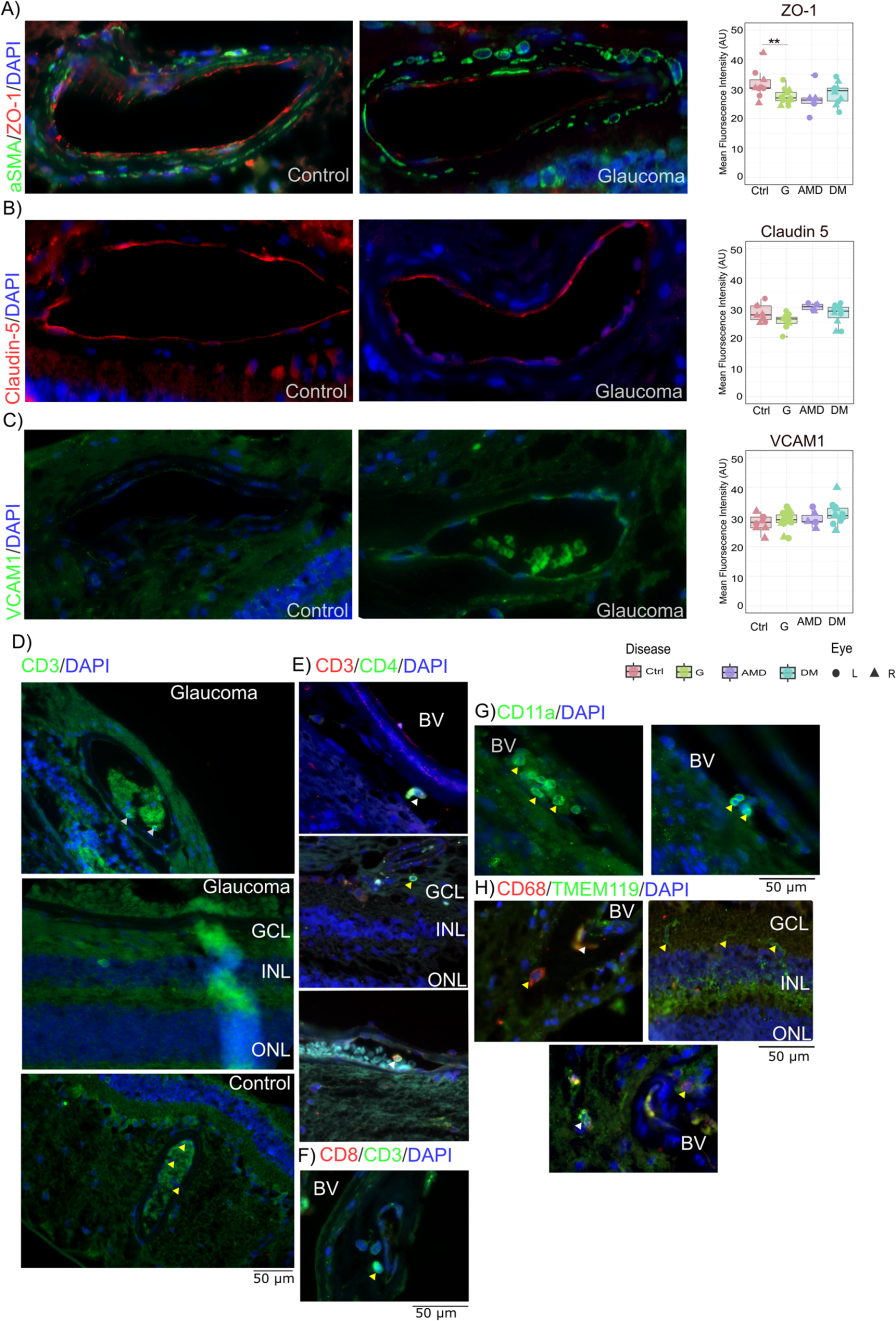
Blood–retinal barrier integrity disruption and immune infiltration. The integrity of the blood‒retinal barrier was assessed via tight junction proteins. (**A**) Representative images showing ZO-1 staining in glaucoma and control samples, with a boxplot visualizing the trend of ZO-1 staining in the sample groups. There was a significant decrease in the ZO-1 staining intensity in the glaucoma samples (**B**). Representative images showing claudin-5 staining in glaucoma and control samples, with a boxplot visualizing the trend of claudin-5 staining in the sample groups. There was a small but insignificant decrease in Claudin-5 in the glaucoma samples. (**C**) Representative images showing VCAM1 staining in glaucoma and control samples, with a boxplot visualizing the trend of VCAM1 staining in the sample groups. The increase in VCAM1 staining intensity did not persist in all the blood vessels in the samples. Representative images showing the presence of CD3 + cells (**D**) in the blood vessels and retina. However, CD3+ cells were rarely observed. Representative images showing the presence of CD3/CD4 + cells (**E**) and the absence of CD3/CD8+ cells (**F**). (**G**) Representative images showing the presence of CD11a+ cells in blood vessels in the retinas of patients with glaucoma. Representative images showing the presence of (**H**) CD68/TMEM119+ cells in the retinas of patients with glaucoma. The presence of systemic immune cells in the glaucomatous retina indicates a dynamic interaction across the blood‒retinal barrier. Abbreviations: AMD = age-related macular degeneration; DM = diabetes; GCL = ganglion cell layer; INL = inner nuclear layer; ONL = outer nuclear layer; Ctrl = control; G = glaucoma. Symbol Means: * = *P* ≤ 0.05; ** = *P* ≤ 0.01; *** = *P* ≤ 0.001; **** = *P* ≤ 0.0001

To assess the state of the BRB, we examined the tight junction proteins, Zonula Occludens-1(ZO-1) and Claudin 5, in the Ctrl and G. We observed a statistically significant decrease in ZO-1 staining (Ctrl: Mean: 32.11 AU, 95% CI: 29.52–34.70; G: Mean: 26.97 AU, 95% CI: 25.75–28.18, *p* = 0.002) (Fig. 4a). In AMD but not in DM, there was also a significant decrease in ZO-1 staining. Although claudin-5 showed a similar decrease (Ctrl: mean: 28.17 AU, 95% CI: 26.42–29.92; G: mean: 25.65 AU, 95% CI: 24.35–26.95; NS) (Fig. 4b), the trend was not statistically significant. We also analysed vascular cell adhesion molecule 1(VCAM1) staining in the retina and ONH, which showed a small increase in intensity across all disease groups. However, this increase was inconsistent across blood vessels in a single sample and was not significant compared with that in Ctrl (Fig. 4c) (Supplementary Table 8).

We utilized lineage markers, including CD3 for T cells and CD19 for B cells. Notably, CD19 + B cells were absent in the retina, whereas CD3+ T cells were identified in both the retinal layers and vasculature (Fig. 4d). CD3+ T cells were observed outside the blood vessels in only eight samples, with 1–2 isolated CD3+ cells occurring more frequently in G than in Ctrl.

We subsequently examined the presence of CD3/CD4+ helper T cells and CD3/CD8+ cytotoxic T cells. CD3/CD8 + cytotoxic T cells were not observed. CD3/CD4+ cells were infrequently identified within the retinal blood vessels of six samples, and only three samples exhibited CD3/CD4+ cell infiltration into the retina (Fig. 4e and f).

Additionally, CD11a+ cells were identified within the blood vessels in some glaucoma samples (Fig. 4g), although these cells were not detected in all patients. To further differentiate resident microglia from infiltrating monocytes and macrophages, we stained for CD68 and TMEM119. Overall, CD68+ cells were sparse (2–3 cells/section) in the glaucoma samples. A subset of cells was positive for CD68 but not TMEM119 and was predominantly located at the optic nerve head ONH and adjacent to blood vessels, with no presence in the retinal layers (Fig. 4h).

## Discussion

Inflammation has been proposed to play a key role in glaucoma progression [30], yet studies investigating glial responses, BRB disruption, and immune cell infiltration in humans are scarce. To address this, we analysed postmortem retinal tissues from glaucoma patients, assessed RGC loss, ONH changes, gliosis, BRB integrity, and immune cell infiltration, and compared them to findings in DM and AMD patients.

Our results confirmed significant RGC loss in G with modest yet significant axonal damage, as measured by reduced β-3-tubulin staining and changes in the ONH, such as increased collagen IV. We also observed an increase in the activity of microglia, astrocytes, and Müller cells, which was marked by increased staining of Iba1, GFAP, and vimentin, respectively. These features helped distinguish G from Ctrl. The extent of RGC loss and axonal damage suggested that our samples likely represented mild-to-moderate disease stages. Notably, gliosis did not correlate with the extent of RGC loss, implying that glial activation may progress independently of neuronal degeneration. This finding might indicate that gliosis is not solely a secondary consequence of RGC death but may actively contribute to disease progression. Moreover, the inability to classify samples based on disease severity may be attributed to the limitations imposed by the small sample size and patient-to-patient variability, which could have influenced the current analysis.

In G, macroglial activation was evidenced by increased GFAP and vimentin staining intensity and expanded cellular area compared with those in the Ctrl group. However, unlike microglia, macroglia (astrocytes and Müller cells) are not directly involved in inflammatory processes. Unlike that reported by Yang et al. 2001 [31] HLA-DR, a marker commonly associated with antigen presentation and inflammatory activation, was localized to Iba1-stained microglia but not astrocytes. These findings suggest that while astrocytes are reactive, their role in glaucoma may be more indirect. Further, it could be speculated that they influence immune response through modulation of the retinal microenvironment and metabolic support rather than through direct participation in immune responses. Indeed, astrocytes have been implicated in maintaining metabolic homeostasis and may exhibit polarization in disease states [31–33]. However, our primary focus on microglial activation led us to use GFAP and vimentin to capture overall macroglial activation. Nonetheless, these findings underscore the need for future studies to comprehensively characterize macroglial functions, particularly metabolic regulation and polarization, in the context of glaucoma pathogenesis.

Microglia, the retina’s primary immune cells, were the focus of our study. Despite their dual roles in maintaining homeostasis and responding to stress, few human studies have explored their activation and functional profiles in glaucoma [20]. Iba1 was used to identify microglia. An increase in both the number of Iba1 + cells and the staining intensity indicated activation of microglia in glaucoma tissues. Additionally, we observed greater diversity in microglial morphology, further reinforcing the concept of microglial activation in glaucoma. These results confirm the presence of microgliosis in glaucoma, which is consistent with prior reports in the literature [6, 32, 34]. In glaucoma, microglia exhibit a shift from a ramified morphology to other morphologies that are associated with microglial activation, such as hyperramified, bushy or ameboid morphology. Hyperramified microglia are associated with early stages, whereas bushy and ameboid microglia are associated with the active defense mode [29]. Therefore, the coexistence of these microglia suggests a continuum of activation. However, given that Iba1 and morphology alone do not confirm functional states, we further characterized microglia using additional markers to better define their activation status.

Our findings collectively indicate that glaucoma is associated with increased immune activity, increased microglial reactivity, and a shift in microglial morphology. To better understand microglial states, we analysed a panel of markers, including TMEM119, CD11b, CD45, HLA-DR, CD68, and CD163. Elevations in CD45 and CD11b, which are commonly used as markers of microglial activation, suggest an intensification of immune responses in the retina. CD45, a pan leukocyte marker, is typically upregulated on activated microglia and other immune cells, indicating an overall increase in immune activity. Similarly, CD11b, a marker of macrophages and microglia, plays a crucial role in the adhesion and migration of immune cells and is elevated in activated microglia, reflecting a potential shift from surveillance to more active immune responses. The increase in CD163, which is associated with anti-inflammatory, reparative macrophages and microglia, further complicates this picture. Changes in CD163 expression may indicate a shift in the functional state of microglia, perhaps towards a more reparative role or a transition between proinflammatory and anti-inflammatory states.

Rutigliani et al. [19]. observed increased Iba1+ microglia/macrophages but low CD45 or CD163 staining, possibly due to their focus on advanced disease stages. Margeta et al. [18] identified increased CD163+ macrophages with minimal CD68+ cells, emphasizing the need for costaining with TMEM119 to differentiate resident microglia from infiltrating macrophages.

Our study is the first to use double immunostaining to comprehensively profile microglia in glaucoma. We found that 87.6% of Iba1+ cells were TMEM119-positive, likely resident microglia, whereas the remaining TMEM119-negative cells may represent infiltrating macrophages. Costaining with CD68 and TMEM119 revealed that a small number of cells expressed only CD68, which may indicate infiltrating macrophages. However, most cells were positive for both CD68 and TMEM119, suggesting that resident microglia are the primary cells that exhibit phagocytic activity, with some influence from infiltrating cells. Some studies also suggest that TMEM119 is downregulated in reactive microglia [35]. This makes it difficult to distinguish TMEM119-positive microglia from infiltrating macrophages. Furthermore, 20% of Iba1 + cells coexpressed CD163, identifying a subset of microglia with potential reparative functions. These findings further underscore the coexistence of microglia in different functional states in glaucoma. While microglia, like macrophages, have traditionally been categorized into M1 (proinflammatory) and M2 (anti-inflammatory) phenotypes, recent studies have criticized this classification as oversimplified [36]. Instead, emerging evidence supports the idea that microglia exist on a continuum of polarization states [37], a concept that is consistent with our findings. Taken together, our results highlight the complexity of microglial activation in glaucoma, revealing a continuum of activation states that include both resident microglia exhibiting phagocytic activity and infiltrating macrophages. These findings challenge traditional M1/M2 classifications and underscore the need for a more nuanced understanding of microglial behavior in glaucoma.

Interestingly, distinct glial response patterns are observed among retinal diseases, reflecting their different aetiologies. This was further supported by the clustering results. For example, AMD involves chronic degeneration of the macula and does not involve RGC loss as a primary feature [38]. Indeed, we did not observe any significant changes in RGC numbers. However, we noted a substantial increase in microglial activity, suggesting an inflammatory component in AMD pathology. Chronic inflammation and drusen accumulation are likely key drivers of microglial activation in AMD, as microglia respond to these persistent stimuli to regulate tissue remodelling and immune surveillance [39]. However, the samples included in our study might constitute the early stages of the disease based on microscopic assessment. This could indicate that gliosis might persist even before geographic atrophy or choroidal neovascularization.

Moreover, DM causes retinal damage due to prolonged hyperglycemia [40]. We observed a prominent increase in macroglia activation, whereas microglial activation was less prominent. The engagement of macroglia in DM can be attributed to early changes in the neuronal changes that precede the overt vascular changes associated with diabetic retinopathy [41]. These cells play crucial roles in maintaining vascular integrity, managing oxidative stress, and impeding vascular damage. The observed divergence in glial activation states—microglial predominance in AMD and macroglial activation in DM—highlights the unique pathophysiological mechanisms underlying these diseases. In AMD, microglial activation is driven primarily by retinal degeneration and stress-related factors, whereas in DM, astrocyte and Müller cell activation can drive vascular damage and metabolic dysregulation, primarily influencing the progression to diabetic retinopathy. Moreover, in glaucoma patients, glial activation is more widespread, with RGC loss. These distinctions in glial behavior are not only indicative of disease-specific mechanisms but also offer potential therapeutic targets. Nonetheless, we did observe some overlap in the clustering. This could be attributed to patient-to-patient variability in disease severity and small sample sizes.

Neuroinflammation in glaucoma also involves infiltrating immune cells. Despite immune privilege due to the BRB, studies in animal models have demonstrated the infiltration of blood-derived cells into the retina upon stimulation [13, 14, 16, 42]. However, studies using human tissues are limited. In our study, we provide evidence of BRB dysregulation, as demonstrated by a decrease in the expression of tight junction proteins such as ZO-1 and Claudin-5 and an increase in the expression of migration-related markers such as VCAM1 and CD11a. ZO-1 is a scaffolding protein that helps connect trans membrane proteins like Claudin-5 to the actin cytoskeleton. Thus, making them integral to maintaining the barrier integrity. Claudin-5 are trans membrane proteins that form the tight junctions; changes to Claudin-5 has been associated with changes to permeability of the retinal barrier [43]. Modest changes to these tight junction proteins could indicate changes to permeability to the BRB rather than complete disruption in glaucoma. VCAM1 is an adhesion molecule expressed on endothelial cells in response to inflammatory cytokines, facilitating immune cell adhesion and migration. Additionally, we observed a few cells expressing CD11a, another adhesion marker found on migrating immune cells. Although inconsistent VCAM1 staining across all the blood vessels in a retina section indicates localised activation of VCAM1. These findings could indicate that tight junction protein dysregulation, and endothelial activation facilitate leukocyte recruitment and immune infiltration in glaucoma. Additionally, activated microglia might influence tight junctions in blood vessels [44]. Immune infiltration is another key feature of neuroinflammation, where systemic cells enter retinal tissue through a compromised barrier [45]. We observed CD3-stained cells in the retina, although their presence was rare and not consistently observed across all samples. We identified CD3/CD4 + helper T cells but not CD3/CD8 + cytotoxic T cells. CD3/CD4 + T cells might either induce microglial activation to a proinflammatory state [46] or dampen the inflammatory response on the basis of their activation states and phenotypic subtypes [47]. These findings indicate that the changes to the permeability of BRB facilitates interactions across the barrier with low incidences of immune infiltration. This highlights the need for further studies to gain deeper insights into the immune cell profile within the retinal microenvironment.

There are several limitations to this study. The use of postmortem retinal tissues prevents the dynamic progression of inflammation, glial activation, and immune cell infiltration. Additionally, the lack of clinical data, such as disease severity, duration, or treatment history, restricts correlations with specific disease stages, as varying disease severity may lead to different stages of glial activation. Variability in immune cell infiltration, particularly inconsistent T-cell presence, complicates interpretation, suggesting individual or disease-related differences in immune responses. Furthermore, the cross-sectional nature of postmortem studies limits the ability to assess temporal changes in immune activation. The sample size and heterogeneity of postmortem tissues may also introduce variability, affecting the generalizability of the findings. Additionally, the absence of in vivo data on retinal function, such as visual acuity or electrophysiological measurements, limits the ability to correlate structural changes with functional outcomes and may not fully reflect the disease’s impact on visual performance.

## Conclusions

In conclusion, this study presents strong evidence highlighting the significant involvement of glial activation and immune cell infiltration in glaucoma. There was widespread activation of glial cells in the glaucoma retina. Moreover, the absence of correlation between glial marker expression and RGC counts suggests that gliosis may not be strictly dependent on the extent of RGC loss and could represent an autonomous response contributing to disease progression. In addition, whether the observed glial changes extend across different stages of glaucoma remains to be determined in future studies with broader clinical data.

**Fig. 5:**
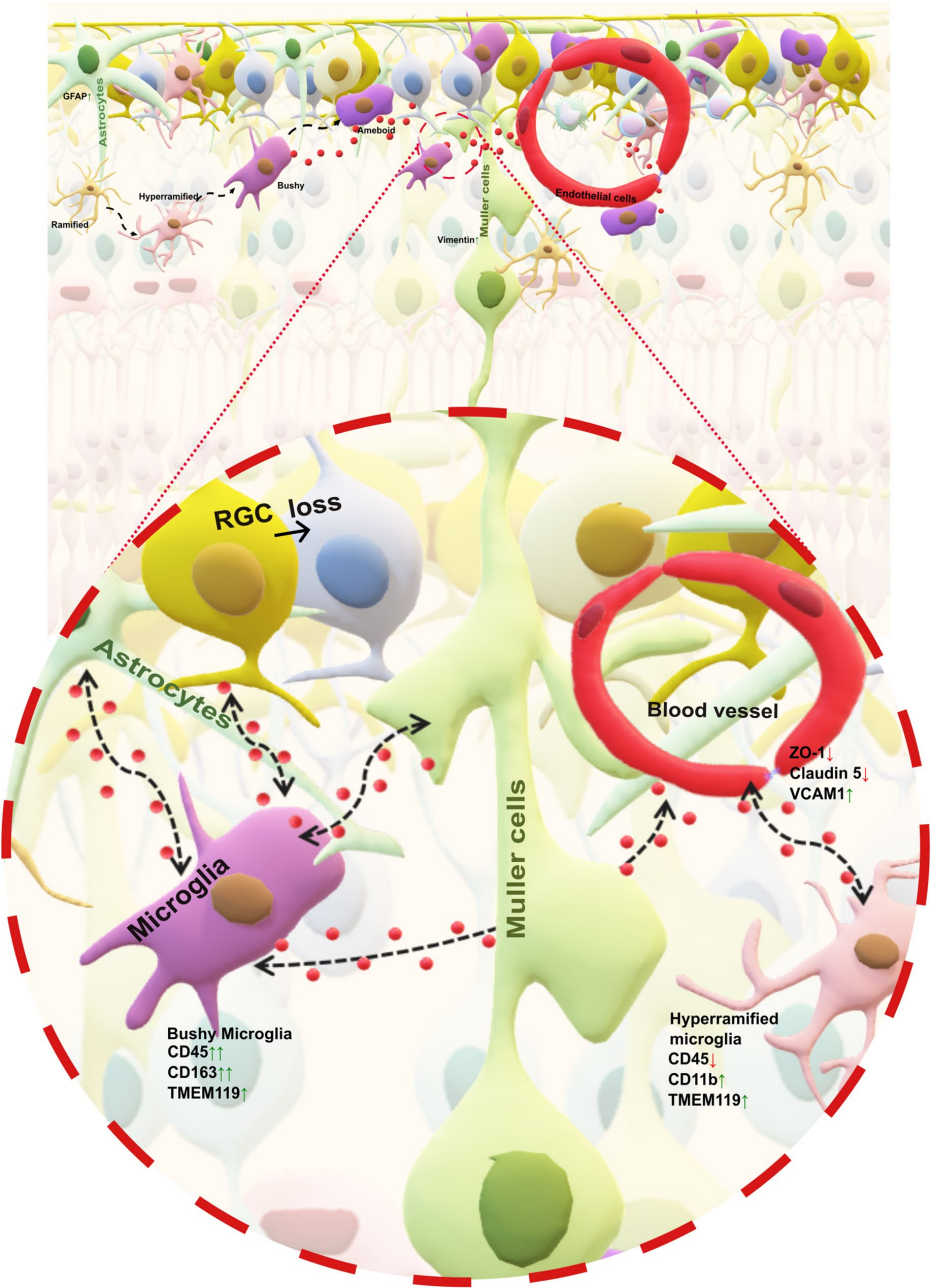
Schematic representation of our findings and derived hypotheses. Glial activation, marked by increased Iba1 (microglia), GFAP (astrocytes), and vimentin (Müller cells), was independent of RGC loss, indicating early gliosis. Microglia exhibit increased numbers, morphological diversity, and functional activation (shift from ramified to activated forms). Most Iba1 + cells were resident microglia (TMEM119+), with a small number of infiltrating macrophages (CD68+). BRB disruption was evident through reduced tight junction proteins (ZO-1 and Claudin-5) and increased VCAM1, suggesting endothelial activation and immune recruitment. Immune cell infiltration was rare, with few CD3 + T cells (mostly CD4+). These findings suggest that resident microglia are the primary mediators of inflammation rather than simply responding to RGC loss. Activated microglia may influence RGC fate through direct interactions with RGCs, glial–glial signalling, and modulation of BRB integrity via tight junction proteins, thereby increasing interactions across the BRB without necessarily promoting immune infiltration. However, these speculations need further investigation

The assessment of the tight junction proteins indicates changes to the permeability of the retina rather than complete disruption of the barrier. In addition, localized activation of cell adhesion molecules points to a microenvironmental influence on barrier regulation. Although immune infiltration is infrequent, it is detectable across different regions. Overall, we could conclude that the resident microglia are primary drivers of the inflammatory responses. The systemic immune involvement might be limited to the exchange of interactor molecules with low incidences of immune infiltration across the BRB (Fig. 5).

However, further research is needed to fully characterize the immune cells involved. Future studies should focus on unravelling glial interactions within the retinal microenvironment via more comprehensive experimental approaches, including in vitro and in vivo models, single-cell transcriptomics, and spatial imaging techniques. Investigating microglia and astrocyte polarization, immune cell dynamics, and cytokine signalling will provide critical insights into the neuroinflammatory processes that drive disease pathology. Additionally, integrating clinical data with molecular profiling could identify biomarkers for early detection and disease monitoring. Exploring therapeutic strategies targeting glial activation, immune modulation, and neuroprotection may also pave the way for novel, personalized treatments for glaucoma.

## Electronic supplementary material

Below is the link to the electronic supplementary material.

Supplementary Material 1

## Data Availability

The data that support the findings of this study are available from the corresponding author upon reasonable request.

## Declarations

### Use of generative AI

During the preparation of this work, the authors used ChatGPT to rephrase some parts of the manuscript to increase readability and clarity. After using this tool/service, the author reviewed and edited the content as needed and took full responsibility for the content of the publication.

### Meeting presentation

Some of these data will be presented at ARVO 2025, 4-8th May 2025, titled “Alterations in Blood‒Retinal Barrier Integrity and Immune Cell Infiltration in Glaucoma: Evidence from Postmortem Tissues.”

## Abbreviations

AMD: Age-related Macular Degeneration
AU: Arbitrary Units
BRB: Blood–Retinal Barrier
CD: Cluster of Differentiation
CI: Confidence Interval
Ctrl: Control
DM: Diabetes Mellitus
ECM: Extracellular Matrix
FFPE: Formalin-Fixed Paraffin-Embedded
GCC: Ganglion Cell Complex
GFAP: Glial Fibrillary Acidic Protein
G: Glaucoma
GCL: Ganglion Cell Layer
GEEs: Generalized Estimating Equations
HLA-DR: Human Leukocyte Antigen– DR isotype
Iba1: Ionized Calcium-Binding Adapter Molecule 1
INL: Inner Nuclear Layer
IOP: Intraocular Pressure
M: Mid-Periphery
ONH: Optic Nerve Head
ONL: Outer Nuclear Layer
P: Periphery
PBS: Phosphate-Buffered Saline
RBPMS: RNA-Binding Protein with Multiple Splicing
RGC: Retinal Ganglion Cell
ROI: Region of Interest
SD: Standard Deviation
SPSS: Statistical Package for the Social Sciences
TMEM119: Transmembrane Protein 119
VCAM1: Vascular Cell Adhesion Molecule 1
ZO-1: Zonula Occludens-1
