# Supplementary material for: Glial and immune dysregulation in glaucoma independent of retinal ganglion cell loss: a human post-mortem histopathology study
Source: Acta Neuropathol Commun. 2025 Jun 28;13:141. doi: 10.1186/s40478-025-02066-0 (PMC12205501; doi:10.1186/s40478-025-02066-0)
Supplement: Supplementary file 1 — Supplementary Material 1 [file 40478_2025_2066_MOESM1_ESM.docx]

**Supplementary Data**

**Independent Glial and Immune Alterations in Glaucoma Beyond Retinal Ganglion Cell Death: Evidence from Human Post-Mortem Analysis**

Akanksha Salkar 1, Viswanthram Palanivel 1, Devaraj Basavarajappa 1, Mehdi Mirzaei1, Angela Schulz1, Peng Yan 3, Vivek Gupta1, Stuart Graham 1, Yuyi You 1,2,*

^1^Macquarie Medical School, Faculty of Human, Health, and Medical Science, Macquarie University. Sydney, NSW, Australia.

^2^Save Sight Institute, University of Sydney. Sydney, NSW, Australia.

3Department of Ophthalmology & Vision Sciences, University of Toronto, Kensington Eye Institute/UHN, Canada.

**
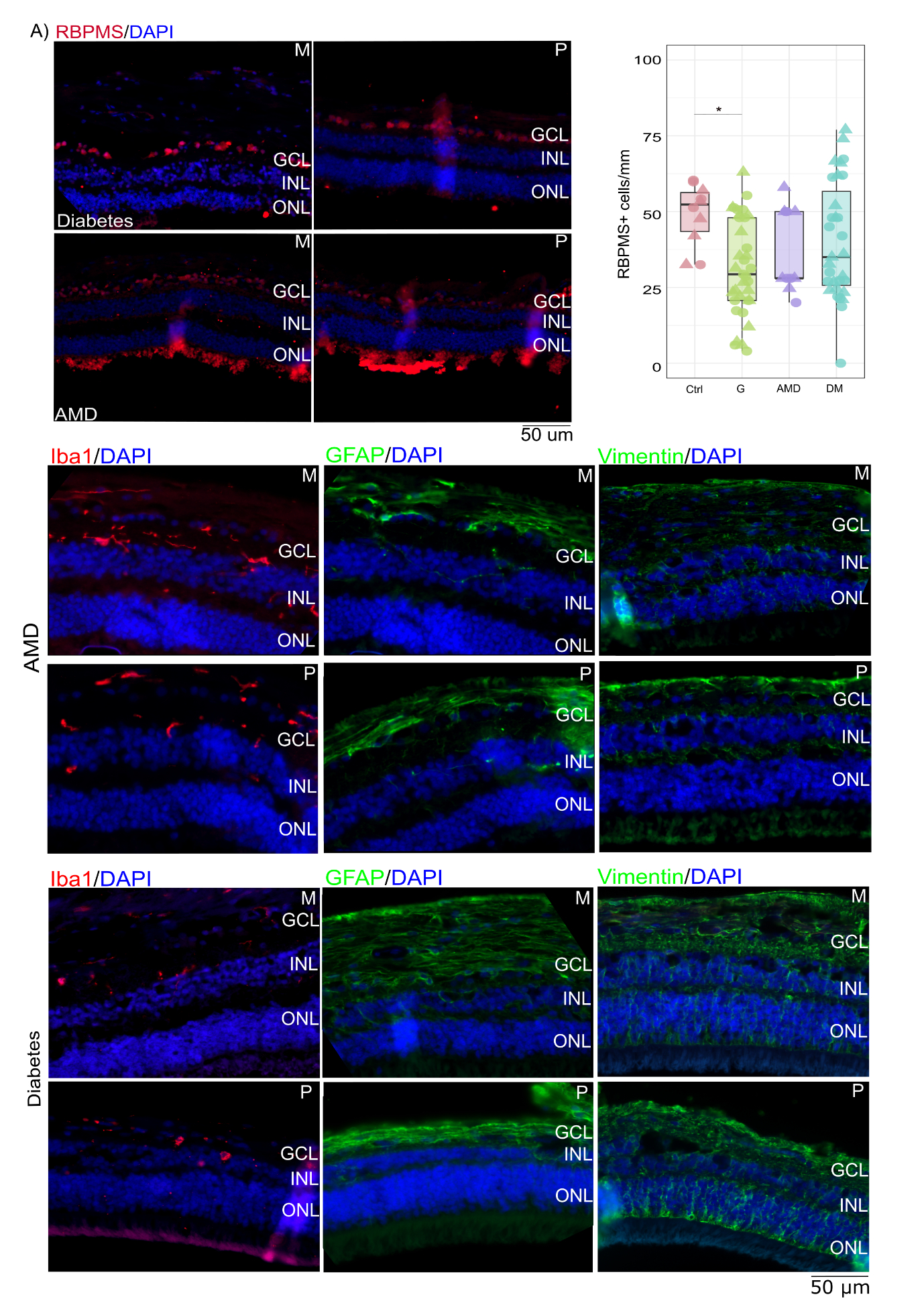
**

**Supplementary Figure 1: RGC counts and glial activity in AMD and DM samples**

A)Representative images showing the RGC stained with RBPMS in AMD and DM retinae (B) Representative images showing Iba1, GFAP, Vimentin staining used as a measure of activation microglia, astrocytes and Muller cells, respectively. *Abbreviations: AMD = age-related macular degeneration; DM = Diabetes; GCL = ganglion cell layer; INL = inner nuclear layer; ONL = outer nuclear layer; Ctrl = Control; G = Glaucoma. Symbol Meaning: * = P ≤ 0.05; ** = P ≤ 0.01; *** = P ≤ 0.001; **** = P ≤ 0.0001*

**
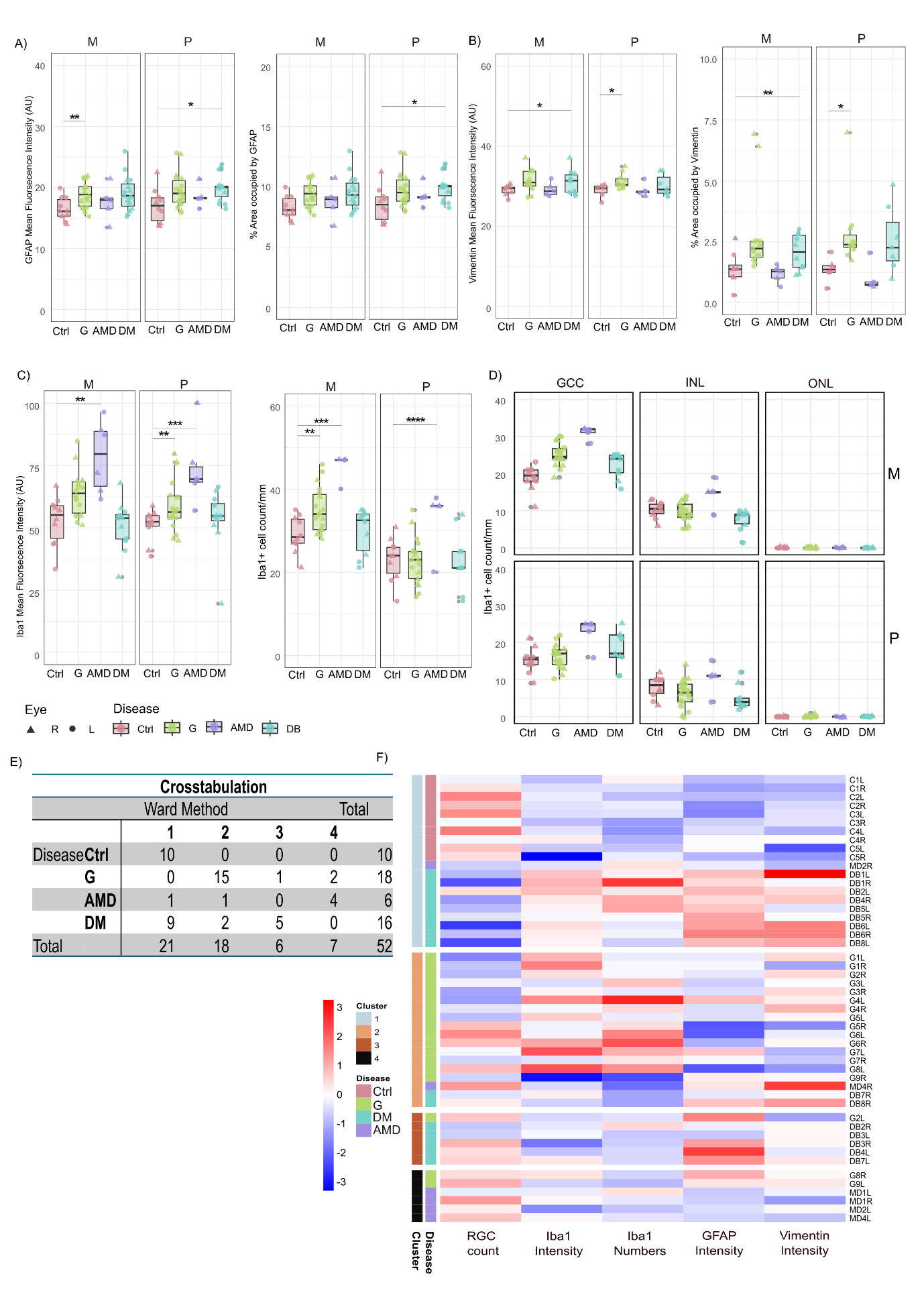
**

**Supplementary Figure 2: Glial activation at the mid-peripheral and peripheral retina**

A) GFAP intensity showed a modest increase in the mid-peripheral retina but was significantly elevated in the peripheral retina of glaucoma and DR samples. A similar trend was observed in % area immunostaining, though not statistically significant. (B) Vimentin staining indicated increased Müller cell activation, with significant elevation in glaucoma and DR samples at the mid-peripheral retina. In glaucoma, activation remained high even in the periphery, with a significant increase in % area occupied by immunostaining. (C) Iba1 intensity increased in glaucoma and AMD samples, with AMD showing heightened microglial activity in both mid-peripheral and peripheral retina, accompanied by an increase in Iba1⁺ cells. (D) In the GCC layer, Iba1⁺ cell numbers were significantly elevated in AMD and glaucoma, particularly in the mid-peripheral retina. Other layers showed increases, though not significant. (E) Hierarchical clustering using RGC counts, Iba1 intensity, microglia count, GFAP intensity, and Vimentin intensity grouped samples based on trends. A heatmap visualizes disease classification by cluster. *Symbol Meaning: * P ≤ 0.05; ** P ≤ 0.01; *** P ≤ 0.001; **** P ≤ 0.0001. Abbreviations: AMD = age-related macular degeneration; GCC = ganglion cell complex; INL = inner nuclear layer; ONL = outer nuclear layer; Ctrl = Control; G = Glaucoma; M=mid peripheral; P=Periphery*

**
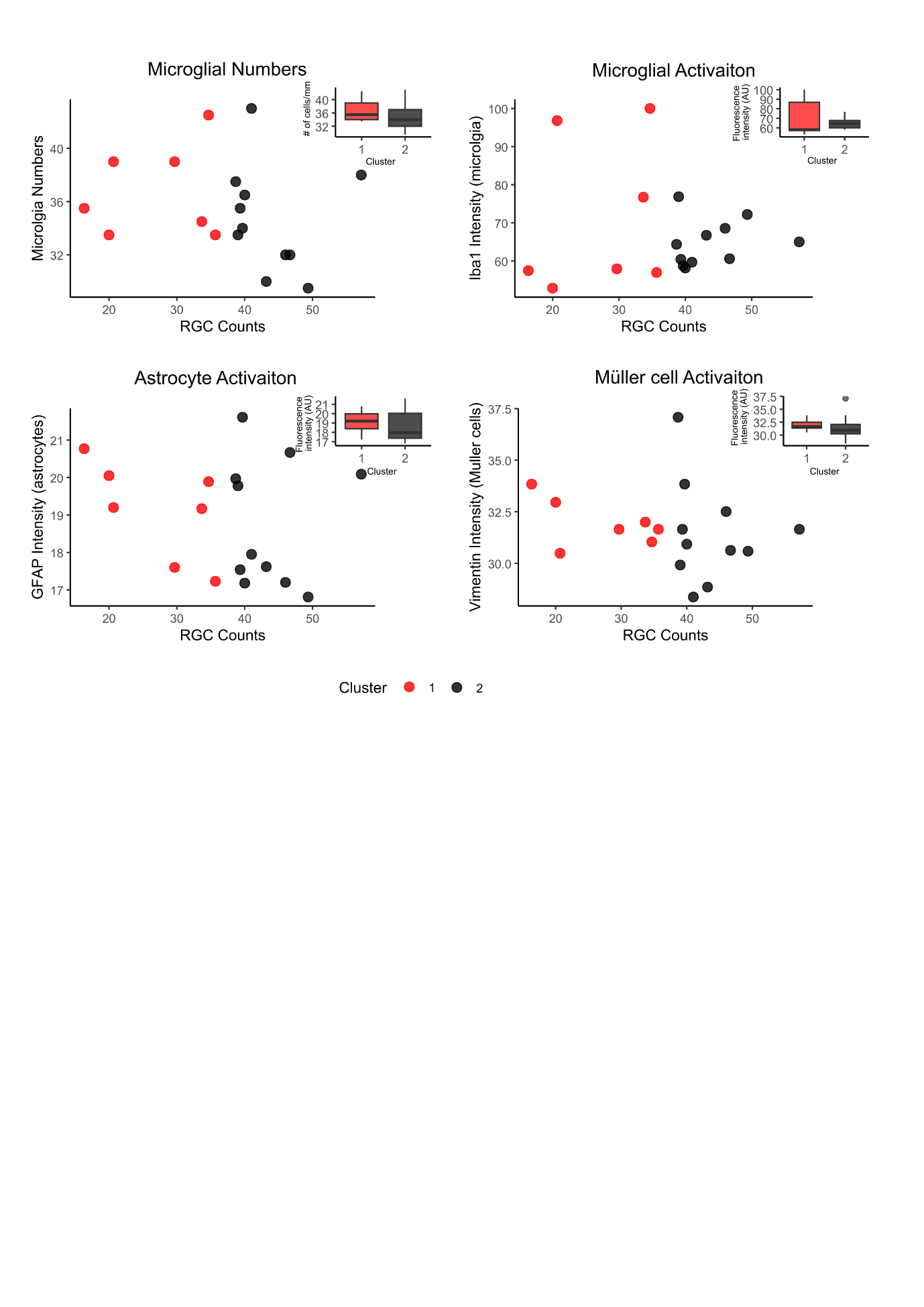
**

**Supplementary Figure 3: Scatterplot of RGC counts vs. glial variables**

The scatter plot visualises the trend in glial variables in the two sample clusters made according to RGC counts. Cluster 1: samples with RGC count less than the average and Cluster 2: samples with RGC count more than the average. *Abbreviations: Ctrl = Control; G = Glaucoma.*

**
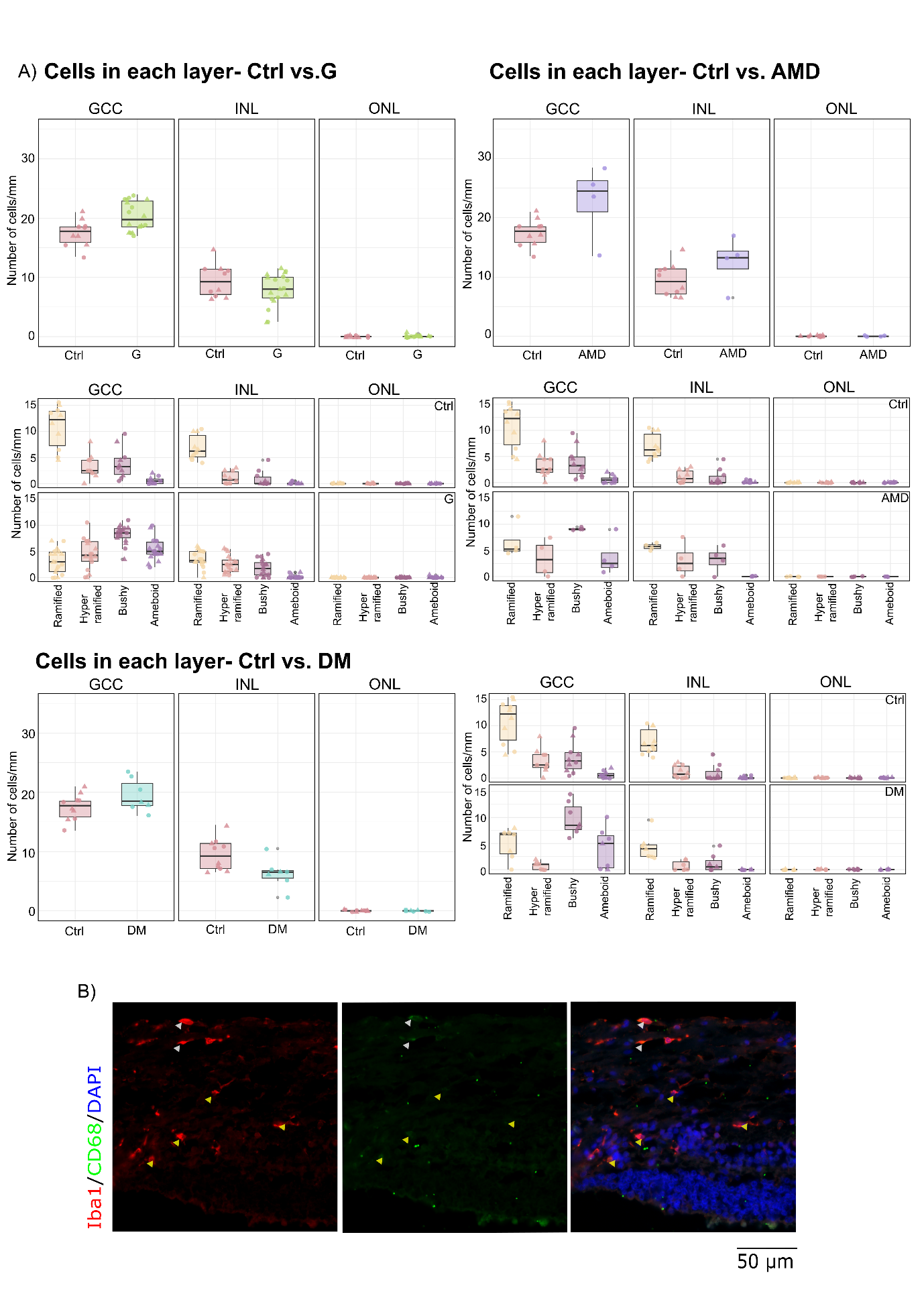
**

**Supplementary Figure 4: Microglial morphological diversity across retinal layers**

A) Boxplot visualising the changes to the microglial number in retinal pathologies compared to control samples across the retinal layers. The boxplot also visualises the morphological diversity across the retinal layer in retinal pathologies. (B) Representative images showing CD68 staining in glaucoma retinae. *Abbreviations: AMD = age-related macular degeneration; DM = Diabetes; GCL = ganglion cell layer; INL = inner nuclear layer; ONL = outer nuclear layer; Ctrl = Control; G = Glaucoma.*

**
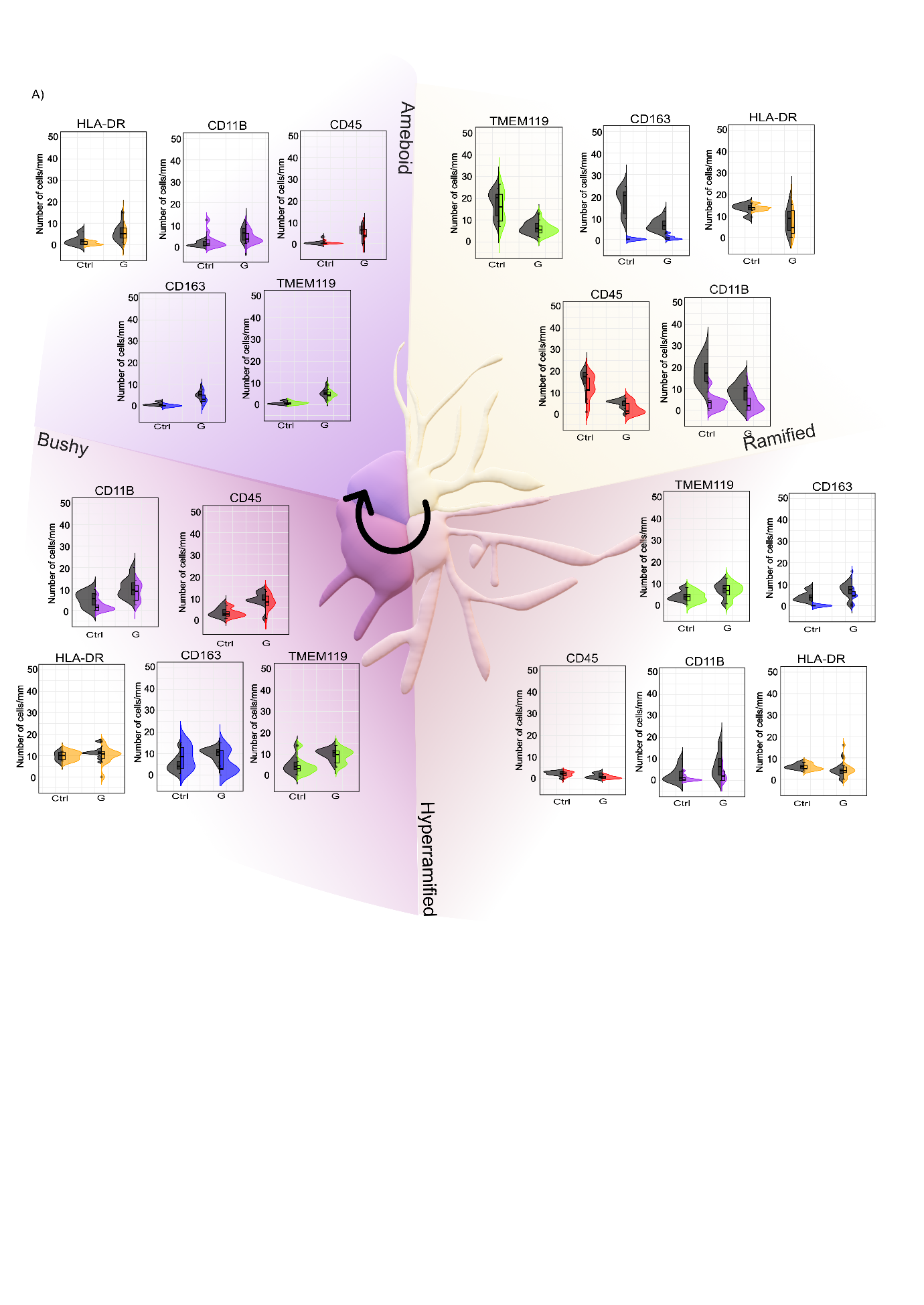
Supplementary Figure 5: Functional marker distribution across the different microglial morphologies**

Split violin plots visualizing the morphology-wise distribution of functional markers such as TMEM119, HLA-DR, CD163, CD68, CD45, and CD11b in Iba1+ve microglial cells. *Abbreviations: Ctrl = Control; G = Glaucoma. Symbol Meaning: * = P ≤ 0.05; ** = P ≤ 0.01; *** = P ≤ 0.001; **** = P ≤ 0.0001*

**Supplementary Figure 1**: List of Antibodies

| **Antibodies** | **Company name** | **Reference number** | **Dilution used** |
| --- | --- | --- | --- |
| **Primary Anitbodies** | | | |
| RGC loss | | | |
| RBPMS | Santa Cruz | sc-293285 | `1:100 |
| beta-3-tubulin | Abcam | ab215037 | `1:200 |
| ONH | | | |
| Collagen IV | Abcam | ab6586 | `1:500 |
| Astrocytes | | | |
| GFAP | Cell Signalling Technology | 3670 | `1:500 |
| Muller cells | | | |
| Vimentin | Abcam | ab92547 | `1:500 |
| Microglia | | | |
| Iba1 | Abcam | ab283319 | `1:500 |
| TMEM119 | Abcam | ab185333 | `1:200 |
| CD45 | Abcam | ab10558 | `1:200 |
| CD68 | Abcam | ab955 | `1:100 |
| CD163 | Abcam | ab182422 | `1:200 |
| HLA-DR | Abcam | ab92511 | `1:200 |
| CD11b | Abcam | ab133357 | `1:100 |
| CD11a | Abcam | ab52895 | `1:200 |
| BRB integrity | | | |
| aSMA | Abcam | ab7817 | `1:500 |
| ZO-1 | Abcam | ab221547 | `1:200 |
| Claudin-5 | Abcam | ab131259 | `1:200 |
| VEGF | Abcam | ab46154 | `1:100 |
| VCAM1 | Abcam | ab134047 | `1:100 |
| Immune infiltration | | | |
| CD3 | Abcam | ab5690 | `1:100 |
| CD3 | Abcam | ab699 | `1:100 |
| CD4 | Abcam | ab133616 | `1:100 |
| CD8 | Cell Signalling Technology | 85336 | `1:100 |
| CD19 | Santa Cruz | sc-8498 | `1:100 |
| **Secondary Antibodies** | | | |
| Alexa Fluor 488-AffiniPure donkey antimouse IgG (H + L) | Jackson ImmunoResearch Labs | AB_2340846 | `1:500 |
| AlexaFlour 647-AffiniPure gost antimouse IgG | Jackson ImmunoResearch Labs |  | `1:500 |
| Cy3-AffiniPure donkey antirabbit IgG (H + L) | Jackson ImmunoResearch Labs | AB_2307443 | `1:500 |
| **Nuclear stain** | | | |
| DAPI |  |  | `1:2000 |

**Supplementary Table 2: RGC loss and other characteristics changes**

| **Parameter** | **Disease group** | **Eccentricity** | **Mean value** | **IQR** | **P VALUE** |
| --- | --- | --- | --- | --- | --- |
| **RGC count**  **(cells/mm)** | Ctrl | Total | 49.07 | 41.68–56.46 |  |
|  |  | M | 59.10 | 46.62–71.58 |  |
|  |  | P | 39.03 | 31.58–46.48 |  |
|  | G | Total | 31.28 | 26.6–36.10 | 0.001 |
|  |  | M | 37.61 | 30.71–44.51 | 0.05 |
|  |  | P | 24.94 | 15.94–33.85 | ns |
|  | AMD | Total | 38.48 | 33.921–43.76 | ns |
|  |  | M | 50.86 | 42.90–58.81 | ns |
|  |  | P | 26.11 | 23.09–29.13 | ns |
|  | DM | Total | 40.69 | 32.10–45.72 | ns |
|  |  | M | 56.90 | 51.38–62.42 | ns |
|  |  | P | 25.99 | 23.93–28.04 | 0.026 |
| **β-III tubulin intensity (AU)** | Ctrl |  | 18.15 | 17.39-18.93 |  |
|  | G |  | 16.49 | 15.98-17.01 | 0.003 |
|  | AMD |  | 19.42 | 18.43-20.42 | ns |
|  | DM |  | 17.84 | 16.72-18.96 | ns |
| **β-III tubulin %area** | Ctrl |  | 12.24 | 10.46-14.03 |  |
|  | G |  | 9.48 | 8.63-10.34 | 0.037 |
|  | AMD |  | 11.53 | 8.74-14.32 | ns |
|  | DM |  | 10.13 | 8.56-11.7 | ns |
| **ColIV intensity (AU)** | Ctrl |  | 18.11 | 16.88-19.34 |  |
|  | G |  | 20.72 | 18.86-22.59 | ns |
|  | AMD |  | 18.23 | 15.17-21.29 | ns |
|  | DM |  | 15.56 | 13.89-17.24 | ns |
| **ColIV %area** | Ctrl |  | 6.51 | 5.43-7.6 |  |
|  | G |  | 10.98 | 8.56-13.41 | 0.006 |
|  | AMD |  | 4.52 | 2.87-6.17 | ns |
|  | DM |  | 3.33 | 2.45-4.22 | 0.0001 |
| Abbreviations: AU=Arbitary units, Ctrl=Control, G=Glaucoma, AMD=age-related macular degeneration, DM=Diabetes, RGC=Retinal ganglion cell, ColIV=collagen IV, M=midperipheral retina, P=Peripheral retina | | | | | |

**Supplementary Table 3:** Glial activation

| **Parameter** |  | **Eccentricity** | **Mean value** | **IQR** | **P VALUE** |
| --- | --- | --- | --- | --- | --- |
| **Iba1 intensity (AU)** | Ctrl | Total | 51.90 | 48.61-55.19 |  |
|  |  | M | 52.73 | 46.55-58.92 |  |
|  |  | P | 51.06 | 48.39-53.74 |  |
|  | G | Total | 62.96 | 56.52–69.41 | 0.016 |
|  |  | M | 67.18 | 58.86-75.51 | ns |
|  |  | P | 58.75 | 52.79-64.71 | 0.009 |
|  | AMD | Total | 75.92 | 72.36-79.5 | 0.0001 |
|  |  | M | 78.52 | 75.92-81.13 | 0.001 |
|  |  | P | 73.32 | 63.73-82.92 | 0.001 |
|  | DM | Total | 52.00 | 46.01-57.99 | ns |
|  |  | M | 50.46 | 45.15-55.78 | ns |
|  |  | P | 53.54 | 46.37-60.71 | ns |
| **Iba1+ve cells (/mm)** | Ctrl | Total | 26.10 | 24.23-27.98 |  |
|  |  | M | 29.10 | 27.85-30.36 |  |
|  |  | P | 23.10 | 19.81-26.4 |  |
|  | G | Total | 28.94 | 26.67-31.23 | ns |
|  |  | M | 35.06 | 32.38-37.74 | 0.02 |
|  |  | P | 22.83 | 20.21-25.47 | ns |
|  | AMD | Total | 37.33 | 33.97-40.7 | 0.0001 |
|  |  | M | 43.25 | 39.67-46.84 | 0.001 |
|  |  | P | 31.42 | 27.61-35.23 | 0.0001 |
|  | DM | Total | 24.82 | 20.34-29.32 | ns |
|  |  | M | 27.36 | 21.2-33.53 | ns |
|  |  | P | 22.29 | 17.89-26.7 | ns |
| **GFAP Mean Intensity (AU)** | Ctrl | Total | 16.74 | 16.18-17.3 |  |
|  |  | M | 16.48 | 15.54-17.44 |  |
|  |  | P | 16.99 | 15.41-18.59 |  |
|  | G | Total | 19.17 | 18.4-19.95 | 0.0001 |
|  |  | M | 18.83 | 17.95-19.73 | 0.011 |
|  |  | P | 19.50 | 18.4-20.61 | ns |
|  | AMD | Total | 18.05 | 16.76-19.36 | ns |
|  |  | M | 17.56 | 15.27-19.85 | ns |
|  |  | P | 18.55 | 18.11-19 | 0 |
|  | DM | Total | 19.47 | 18.52-20.43 | 0.0001 |
|  |  | M | 19.05 | 17.31-20.79 | ns |
|  |  | P | 19.90 | 19.01-20.8 | 0.05 |
| **Vimentin Mean Intensity (AU)** | Ctrl | Total | 28.89 | 27.86-29.94 |  |
|  |  | M | 28.93 | 27.97-29.89 |  |
|  |  | P | 28.86 | 27.47-30.26 | . |
|  | G | Total | 31.40 | 30.55-32.27 | 0.002 |
|  |  | M | 31.10 | 30.06-32.14 | ns |
|  |  | P | 31.71 | 30.65-32.79 | 0.041 |
|  | AMD | Total | 29.11 | 27.63-30.59 | ns |
|  |  | M | 29.19 | 27.49-30.9 | ns |
|  |  | P | 29.03 | 27.78-30.29 | ns |
|  | DM | Total | 31.07 | 29.77-32.38 | ns |
|  |  | M | 31.93 | 30.39-33.48 | 0.034 |
|  |  | P | 30.21 | 28.65-31.79 | ns |

Abbreviations: Ctrl=Control, G=Glaucoma, AMD=age–related macular degeneration, DM=Diabetes, RGC=Retinal ganglion cell, ColIV=collagen IV, M=Mid-peripheral retina, P=Peripheral retina.

**Supplementary Table 4: Correlation between RGC counts and glial variables in glaucoma samples**

| **Variables** | **Pearson Correlation (r)** | **Sig. (2-tailed)** |
| --- | --- | --- |
| RGC counts - Microglia numbers | 1 | - |
| RGC counts - Microglia numbers | -0.226 | 0.367 |
| RGC counts - GFAP intensity | -0.237 | 0.344 |
| RGC counts - Vimentin intensity | -0.238 | 0.341 |
| RGC counts - Iba1 intensity^b^ | 0.224 | 0.372 |

^b^Spearman's correlation

**Supplementary Table 5**: Changes to the area covered by GFAP and Vimentin staining

| **Parameter** |  | **Eccentricity** | **Mean value** | **IQR** | **P VALUE** |
| --- | --- | --- | --- | --- | --- |
| GFAP %Area covered (%) | Ctrl | Total | 4.37 | 2.98-5.77 |  |
|  |  | M | 5.08 | 2.73-7.44 |  |
|  |  | P | 3.67 | 2.94-4.41 |  |
|  | G | Total | 5.95 | 5.05-6.86 | ns |
|  |  | M | 6.28 | 5.27-7.3 | ns |
|  |  | P | 5.62 | 4.46-6.78 | ns |
|  | AMD | Total | 4.43 | 3.95-4.92 | ns |
|  |  | M | 3.79 | 2.06-5.52 | ns |
|  |  | P | 5.08 | 3.51-6.65 | ns |
|  | DM | Total | 6.76 | 5.88-7.66 | 0.028 |
|  |  | M | 7.05 | 5.96-8.14 | ns |
|  |  | P | 6.48 | 4.88-8.1 | 0.05 |
| Vimentin %Area covered(%) | Ctrl | Total | 1.36 | 0.94-1.78 |  |
|  |  | M | 1.39 | 0.86-1.93 |  |
|  |  | P | 1.32 | 0.99-1.66 |  |
|  | G | Total | 2.91 | 2.14-3.7 | 0.004 |
|  |  | M | 2.97 | 2.06-3.89 | ns |
|  |  | P | 2.85 | 2.08-3.64 | 0.011 |
|  | AMD | Total | 1.07 | 0.79-1.36 | ns |
|  |  | M | 1.16 | 0.87-1.45 | ns |
|  |  | P | 0.98 | 0.61-1.36 | ns |
|  | DM | Total | 2.45 | 1.83-3.07 | 0.026 |
|  |  | M | 2.26 | 1.84-2.69 | 0.018 |
|  |  | P | 2.63 | 1.69-3.58 | ns |

Abbreviations: Ctrl=Control, G=Glaucoma, AMD=age–related macular degeneration, DM=Diabetes, RGC=Retinal ganglion cell, ColIV=collagen IV, M=Mid-peripheral retina, P=Peripheral retina.

**Supplementary Table 6:** Layer–wise distribution of Iba1+ve microglial cells in glaucoma retina

| Disease | Retinal layer | GCC | INL | ONL |
| --- | --- | --- | --- | --- |
| Ctrl | Total | 17(15.49-18.52) | 9.1(8.16-10.05) | 0(0–0) |
|  | M | 18.8(17.43-20.18) | 10.3(9.4-11.21 | 0(0–0) |
|  | P | 15.2(13.12-17.29) | 7.9(6.26-9.55 | 0(0–0) |
| G | Total | 20.58(19.32-21.86)^B^ | 6.29(6.21-9.57)^NS^ | 0.03(0–0.008)^NS^ |
|  | M | 25.12(23.68-26.55)^D^ | 9.62(8.09-11.15)^NS^ | 0(0–0)^NS^ |
|  | P | 16.06(14.45-17.67)^NS^ | 6.17(4.05-8.29)^NS^ | 0.06(0–0.16)^NS^ |
| AMD | Total | 24.67(20.38-28.96)^B^ | 7.89(10.12-15.23)^NS^ | 0(0–0)^NS^ |
|  | M | 28.84(24.17-33.51)^D^ | 14.42(12.4-16.44)^B^ | 0(0–0)^NS^ |
|  | P | 20.5(16.35-24.66)^D^ | 10.92(7.8-14.04)^NS^ | 0(0–0)^NS^ |
| DM | Total | 19.81(17.69-21.95)^NS^ | 6.29(4.75-7.83)^A^ | 0(0–0)^NS^ |
|  | M | 22.19(20.52-23.86)^NS^ | 7.67(5.91-9.43)^NS^ | 0(0–0)^NS^ |
|  | P | 17.45(14.37-20.53)^NS^ | 4.92(2.68-7.16)^NS^ | 0(0–0)^NS^ |

The data is presented as average number of cells/mm (95%CI). Abbreviations: Ctrl=Control, G=Glaucoma, AMD=age–related macular degeneration, DM=Diabetes, RGC=Retinal ganglion cell, ColIV=collagen IV, M=Mid-peripheral retina, P=Peripheral retina, GCC=ganglion cell complex, INL=inner nuclear layer, ONL=outer nuclear layer. Ctrl vs. Disease :A=P ≤ 0.05, B=P ≤ 0.01, C=P ≤ 0.001, D=P ≤ 0.0001

**Supplementary Table 7: Morphology-based distribution of Iba1+ve cells**

| Disease | Total | Ramified | Hyperramified | Bushy | Ameboid |
| --- | --- | --- | --- | --- | --- |
| Control | 26.1(24.23-27.98) | 16.8(13.38-20.23) | 4.3(3.15-5.46) | 4.8(1.89-7.72) | 0.7(0.11-1.3) |
| Glaucoma | 28.94(26.67-31.23) | 6.7(5.22-8.18)^D^ | 7.03(5.06-8.99)^D^ | 10.14(8.86-11.43)^C^ | 5.67(4.52-6.82)^D^ |
| AMD | 37.33(33.97-40.7) | 11.59(9.62-13.55) | 7.42(5.46-9.39)^A^ | 12.25(10.74-13.77)^D^ | 5.46(2.17-8.76)^A^ |
| DM | 24.82(20.34-29.32) | 7.71(5.04-10.37)^D^ | 1.14(0.38-1.9) | 11.88(9.48-14.28)^C^ | 5.13(2.65-7.62)^B^ |

The data is presented as average number of cells/mm (95%CI). Abbreviations: Ctrl=Control, G=Glaucoma, AMD=age–related macular degeneration, DM=Diabetes, RGC=Retinal ganglion cell.

**Supplementary Table 8:** Quantification of changes to the Blood–Retinal barrier components

| **Parameter** | **Disease** | **Mean value** | **IQR** | | **p-value** |
| --- | --- | --- | --- | --- | --- |
| **ZO-1 staining intensity (AU)** | Ctrl | 32.11 | 29.52-34.7 |  | |
|  | G | 26.97 | 25.75-28.18 | | 0.002 |
|  | AMD | 26.49 | 22.73-30.26 | | NS |
|  | DM | 28.51 | 26.03-31 | | NS |
| **Claudin-5 staining intensity (AU)** | Ctrl | 28.17 | 26.42-29.92 |  | |
|  | G | 25.65 | 24.35-26.95 | | NS |
|  | AMD | 30.28 | 29.26-31.29 | | NS |
|  | DM | 27.90 | 26.35-29.45 | | NS |
| **VCAM1staining intensity (AU)** | Ctrl | 27.94 | 26.07-29.82 |  | |
|  | G | 29.06 | 27.76-30.36 | | NS |
|  | AMD | 29.32 | 26.83-31.8 | | NS |
|  | DM | 30.98 | 28.57-33.39 | | NS |

Abbreviations: Ctrl=Control, G=Glaucoma, AMD=age–related macular degeneration, DM=Diabetes, RGC=Retinal ganglion cell, ColIV=collagen IV, M=Mid-peripheral retina, P=Peripheral retina, GCC=ganglion cell complex, INL=inner nuclear layer, ONL=outer nuclear layer
